# Calculation of Disease Dynamics in a Population of Households

**DOI:** 10.1371/journal.pone.0009666

**Published:** 2010-03-18

**Authors:** Joshua V. Ross, Thomas House, Matt J. Keeling

**Affiliations:** 1 King's College, University of Cambridge, Cambridge, United Kingdom; 2 Biological Sciences and Mathematics Institute, University of Warwick, Coventry, United Kingdom; University of Swansea, United Kingdom

## Abstract

Early mathematical representations of infectious disease dynamics assumed a single, large, homogeneously mixing population. Over the past decade there has been growing interest in models consisting of multiple smaller subpopulations (households, workplaces, schools, communities), with the natural assumption of strong homogeneous mixing within each subpopulation, and weaker transmission between subpopulations. Here we consider a model of *SIRS* (susceptible-infectious-recovered-susceptible) infection dynamics in a very large (assumed infinite) population of households, with the simplifying assumption that each household is of the same size (although all methods may be extended to a population with a heterogeneous distribution of household sizes). For this households model we present efficient methods for studying several quantities of epidemiological interest: (i) the threshold for invasion; (ii) the early growth rate; (iii) the household offspring distribution; (iv) the endemic prevalence of infection; and (v) the transient dynamics of the process. We utilize these methods to explore a wide region of parameter space appropriate for human infectious diseases. We then extend these results to consider the effects of more realistic gamma-distributed infectious periods. We discuss how all these results differ from standard homogeneous-mixing models and assess the implications for the invasion, transmission and persistence of infection. The computational efficiency of the methodology presented here will hopefully aid in the parameterisation of structured models and in the evaluation of appropriate responses for future disease outbreaks.

## Introduction

The earliest models proposed for infectious disease dynamics assumed that the population afflicted by the pathogen was large and homogeneously mixed such that deterministic equations with simple frequency-dependent transmission were appropriate [1]–[3]. These models were subsequently extended in three major directions: taking into account the discrete nature of populations and the stochastic nature of transmission and recovery [4]–[6]; taking into account heterogeneity between individuals in terms of differential mixing [7]; and accounting for spatial structure and the often localized transmission of infection (a variety of approaches are summarised in [3, Chapter 7]). This latter extension has taken several different forms but can be predominately dichotomized into those based upon explicit contact networks that determine the possible opportunities for transmission between individuals [8]–[17] and those models that stratify the population into sub-populations (for example households), with, typically, homogeneous mixing within the subpopulations and weaker mixing between them [18]–[25] (although there exist exceptions, including those that bridge this divide; see for example [26]–[28]). The former network models are most appropriate for situations where there exists explicit knowledge of contact structure heterogeneity such as for sexually transmitted diseases [13], the air transport network for SARS [29] and for livestock movements in Britain [30], [31]. The latter structured or metapopulation models reflect the relatively strong opportunity for transmission between individuals within a household compared to transmission to other individuals in the population. These households models can therefore be conceptualised as a combination of a network model (for the strong within household transmission) and a homogeneous mixing model (for the weaker transmission to the general population).

Household structured models offer an attractive trade-off between fine-scale detail and computational feasibility, and for this reason they increasingly form the basis of studies in disease management. For example, it has been known for some time that household structure influences the critical threshold for invasion and vaccination [20], [32], and more recently ideas from formal modelling have been used to answer increasingly applied questions: these range from parameter estimation [33] to evaluation of an appropriate response to an influenza pandemic [34], [35].

Here we consider a pathogen for which individuals develop transient immunity following infection, with the immunity waning resulting in the individual eventually returning to full-susceptibility to the disease: the *SIRS* (susceptible-infected-recovered-susceptible) model. We model the spread of such a pathogen amongst individuals occupying a very large (assumed infinite) set of households. The infection dynamics within each household is captured by a Markov chain representation, accounting for the stochastic effects due to the small number of individuals within each household. We assume that transmission between households results from (effective) contacts between individuals in the population occurring at a fixed rate. Models of this form have been studied widely recently [21]–[24], [36], [37], although the particular characteristic of waning immunity has received relatively less attention [24], [38]. We then extend this model to additionally incorporate more realistic distributions for the infectious period; this is achieved by using the classical approach of breaking the infectious class into a number of new classes while preserving the total average time in these new classes (see, for example, [39] and [40]).

In this paper we investigate a range of epidemiologically important quantities over a large region of parameter space; as such we present and utilise efficient methods for evaluating these quantities. Some of the methods we adopt have been described elsewhere, and alternative methods exist for evaluating several of the quantities. In particular the classic work on households models such as [21] often includes more general features than we consider here; however as a consequence of this generality, many of the results are not as readily amenable to rapid numerical evaluation. Here we present results and numerical methods appropriate for studying our model, and closely related ones, in a unified and accessible form, hence allowing their direct application to epidemiological problems. For this very reason we provide MATLAB code to evaluate the quantities considered (see Supporting Information File S2); MATLAB provides an ideal language and framework for the manipulation of the transition matrices that are associated with the Markov chain approach adopted here.

Five epidemiologically important quantities are evaluated: (i) the threshold for invasion, 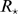, which is the household basic reproduction number [21], which measures the average number of secondary households infected from a single infected household in a totally susceptible population and therefore determines if a pathogen may successfully invade (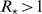); (ii) the early growth rate, 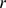, (also called the *Malthusian parameter* [21]), which is the quantity generally measured for statistical inference in the early stages of an emerging infection; (iii) the household offspring distribution, defined as the distribution of secondary households infected from an epidemic seeded with a single infectious individual – the expectation of this random variable is 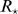; (iv) the endemic prevalence of infection 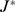 amongst the community of households; and (v) the distribution of transient dynamics of the process. This latter calculation not only provides an opportunity to investigate the impact of the models structure in detail, but provides an alternative method to assess the accuracy of the many quantities listed above.

In the next section we introduce the basic model of within- and between-household transmission, before detailing the methodology adopted, and illustrate several of the techniques with respect to a model for households of size one (equivalent to the classical homogeneously-mixing *SIR* model). We then proceed to apply this methodology to our structured households models to investigate the impact of household size, within- and between-household transmission rates, rate of waning immunity, and infectious period distribution on dynamics. We conclude by discussing the general implications of our results.

## Methods

The household dynamics are described by three basic processes: transmission of infection between an infectious and susceptible individual within the household, recovery of infected individuals, and loss of immunity (Table 1). Recovery of an infected individual and loss of immunity for a recovered individual are both assumed to occur independently of the states of other individuals within the household with constant probabilistic rates 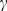 and 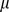, respectively. Transmission within the household is assumed to be frequency dependent with transmission parameter 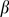; note that the 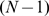 term in the denominator is to ensure frequency dependent contact with all other members of the household. In situations where we need to consider the transient or long-term dynamics it becomes important to allow infection to enter the household from the external population. This is captured by an external force of infection 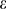 to all susceptible individuals within the household. Finally, we assume a between-household transmission rate 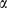, such that each infectious individual within the household generates secondary cases at rate 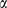. Clearly, for self-consistent dynamics we insist that 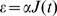, where 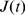 is the proportion of infectious individuals in the population.

**Table 1:** Classical within-household *SIRS* model of epidemic dynamics.

Event	Transition	Rate
Internal Infection	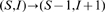	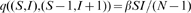
Recovery	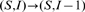	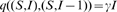
Waning Immunity	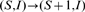	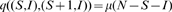
External Infection	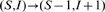	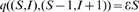

The first four quantities of interest (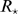, 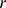, distribution of secondary households, and the prevalence of infection) can all be efficiently evaluated by solving systems of linear equations (for example, using the backslash operator
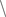
in MATLAB). In particular, these quantities are based upon the expectation, or distribution, of a path integral of a Markov chain [41]–[43]; we therefore present the necessary aspects of this theory, for the most part taken from Pollett and Stefanov [42].

### Expectation and distribution of path integrals for Markov chains

Let 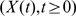 be a continuous-time Markov chain taking values on a finite subset of the non-negative integers 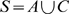, where 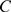 is a set of transient states and 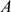 is a set of absorbing states, so the chain is absorbed almost-surely in finite time. (For our household dynamics, when 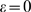, 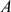 refers to when the entire household is susceptible and 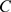 is the set of all other (transient) states for a household). Now consider a function 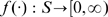 with 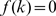 for 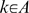; 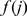 may be thought of as a per-unit reward when in state 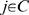 or for the households model can be naturally used to count the number of infected individuals in a given household configuration. Now, consider the path integral
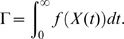
This path integral is the total reward over the life of the process, and as such is a random variable. We now present systems of linear equations for evaluating the expected value and Laplace-Stieltjes transform of the distribution of 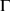.

The behaviour of the continuous-time Markov chain can be defined by fixed transition rates between states which we formulate into a matrix 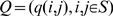, with 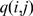 representing the rate of transition from state 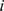 to state 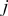, for 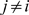, and 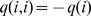, where 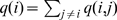 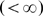, is the total rate at which the process leaves state 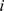.

The expected value of 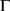 conditional on starting the process in state 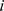, namely 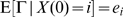, may be determined by the solution of the system of linear equations (Proposition 2 [42]):
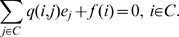
(1)Furthermore, letting 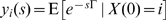 (the Laplace-Stieltjes transform of the distribution of 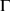) and noting 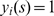 for 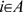, we have [42, Proposition 1]: For each 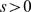, 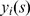 is the solution of the system of linear equations:
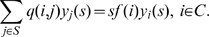
(2)Using these two sets of linear equations it is possible to efficiently evaluate a number of quantities concerning the early dynamics of infection within structured households models.

### Households of size one

To illustrate the power of this methodology, we consider the dynamics of households of size one with *SIR* dynamics for which many of the key epidemiological quantities are already known. (This can also be realised by setting the within-household transmission rate, 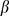, to zero, but the computation is more complex.) Assuming that the number of households is infinite, then an initial infectious individual creates a new infectious household (individual) at rate 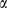 over the course of their infectious lifetime, and moves from the infected to recovered state at rate 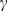. The Markov process for within-household dynamics can be formulated by letting 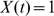 if the individual is infectious, and 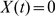 if it has recovered; we therefore have 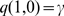 (and thus 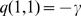).

We are naturally interested in onward transmission from this household, which means that we wish to consider 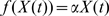. Then, from (1), the expected number of secondary households infected by this infectious individual over its lifetime, 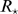 (in this case equivalent to 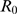) is the solution 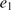 to: 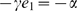. Hence 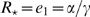 as expected from fundamental results for the *SIR* model.

Now, for future use, consider the distribution of the path integral. We have from (2): 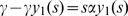, and thus 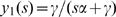, and upon inverting this Laplace transform we find that 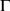 is exponentially distributed with mean 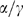 – that is, 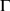 has a probability density function 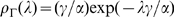. Whilst we will not be interested in this distribution directly *per se*, it does allow us to evaluate the full (discrete) distribution of secondary infections: the offspring distribution is Poisson with random mean, and the probability density function of the mean is 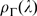 [18], [44]. Thus, the probability mass function of secondary infections, 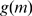 (the probability that an infected individual generates 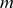 secondary cases), for any integer 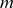 is given by
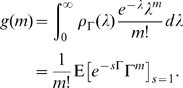
We note here that we may substitute 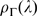 and integrate to obtain
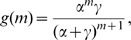
which is the geometric distribution with parameter 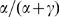, as expected from the basic dynamics. However, by noting the close resemblance to the Laplace-Stieltjes transform introduced earlier, we develop an alternative procedure which is in general more efficient. Consideration of the expectation component gives
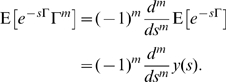
Hence, 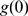 may be determined as the solution of the system of linear equations (2), as 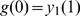. Then, differentiating the system (2), we have
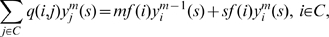
(3)where 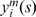 denotes the 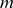
th derivative of 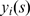, allowing us to recursively evaluate the distribution of secondary infections from
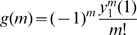
(we may stop the recursive evaluation once the cumulative probability mass is close to 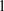). For the case of households of size one, we readily arrive at the geometric distribution with parameter 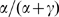, as evaluated earlier by direct integration.

Finally, we consider the early growth rate 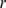 of the epidemic (equivalent to the *Malthusian parameter* of the branching process approximation of Ball *et al.* [21]), defined as the solution to the equation
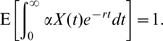
We note that this is again the expectation of a path integral, but with exponential discounting at rate 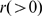; as presented in Norris [41], and easily seen, this is equivalent to the path integral of the original process modified so that from each state 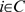 we add a rate 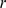 of jumping to the absorbing set 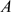. Thus, for households of size one: 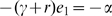, and upon solving 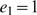 for 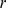 we determine 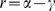, a well-known result from the literature.

### A population of households

We now turn our attention to evaluating each of the desired quantities for households of arbitrary, but homogeneous, size. Populations of mixed household sizes are achievable through the same basic methodology, but the ensuing behaviour is more difficult to visualise and the relationships to the distribution of household sizes is naturally more complex. When dealing with household dynamics we initially consider a Markov chain model of an epidemic occurring within the initially infected household, effectively setting 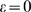, and determine the subsequent dynamics by considering the rate at which subsequent households become infected – mirroring the mechanisms used for households of size one. For simplicity, from now on we rescale time such that the recovery rate 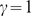; effectively our new unit of time is the average infectious period.

#### (i) Household basic reproduction number, 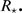


The household basic reproduction number 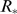 is calculated as the expected number of secondary households infected by the primary household, which naturally scales with the amount of infection within the primary household. We define 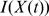 as the function of the underlying household Markov chain that gives the number of infected individuals within the household at time 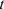. The household basic reproduction number is then given by
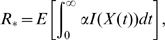
where 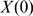 refers to a household with a single infection and the remaining members susceptible [21], [24], [36]. This is calculated as described above, solving the system of linear equations (1). Evaluating 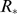 with 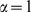 gives us the critical value 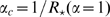, at which 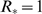 and is therefore the invasion threshold.

#### (ii) Early growth rate

The early growth rate 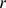 obeys
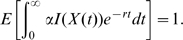
This can be derived either from consideration of the branching process approximations [21], or a survivor model [33], and can be evaluated using exponential discounting as outlined above [41]. Since 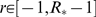 [45] (here we only consider epidemics which invade, and thus 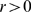) and the expectation monotonically decreases with 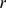, a unique solution exists and can be found numerically using, for example, interval bisection or MATLAB's fzero routine.

#### (iii) Household offspring distribution

The household offspring distribution during the early stages of the epidemic is again Poisson with random mean:
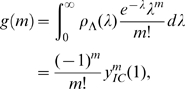
(4)for integer 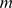 and where 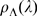 is the probability density function of the stochastic variable
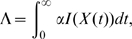


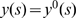
 is the Laplace transform of the distribution of 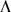, and 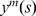 is the 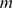-th derivative of this transform with respect to 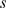. As with the two above quantities, we are interested in the entry corresponding to the initial condition (IC) 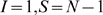. We can calculate the offspring distribution (4) by solving systems of linear equations (2,3), as outlined earlier for the case of households of size one.

#### (iv) Endemic prevalence

To evaluate the endemic prevalence, we could evaluate the full transient dynamics of the process, as explained in the next section, and evaluate the endemic prevalence via convergence to equilibrium of these dynamics. However, it is possible to develop a more efficient procedure, which does not rely on numerically solving the differential equations.

We exploit the fact that at equilibrium the rate of import of infection into a household, 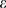, must be equal to the rate of export of infection 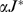. Throughout this paper, we use 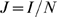 to represent the proportion of individuals infected. Thus, our starting point for determining the endemic proportion of infection 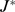 in the population is by considering the dynamics within a single household, given by our within-household Markov chain model detailed in Table 1, with constant (and not yet self-consistent) external force of infection 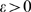. Using an eigenvalue-vector routine, for example the MATLAB function eig, we find the eigenvector corresponding to the zero eigenvalue; normalised to sum to 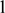, this eigenvalue 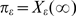 is the stationary (equilibrium) distribution of the within-household epidemic with import at rate 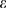. We then search for the 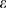 such that 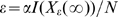, which can be done with considerable efficiency given that 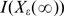 increases monotonically with 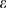. The endemic prevalence 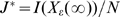.

#### (v) Transient dynamics

To model the full dynamics of the system we need to both include the external rate of infection 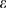 and to allow it to dynamically vary in a self-consistent manner [46]. Let 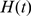 be the vector of proportions of households in each possible disease configuration at time 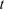; its dynamics are described by the coupled ODEs:
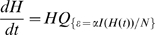
(5)where 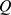 is the household transition matrix together with the dynamically varying external force of infection. We note that this equation may also be motivated by using results of Kurtz [47], considering the proportion of household types in the limit as the number of households tends to infinity. Due to the fact that 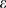 varies during the transient dynamics methodologies based on taking the exponential of matrices [48] are not applicable. The full dynamical system (5) is therefore solved numerically using standard Runge-Kutta methods, for example as implemented in MATLAB's ode45.

#### Additional realism

As noted earlier, we also consider the effect of gamma-distributed infectious periods on several of the above quantities. This is achieved by using the commonly-termed *method of stages*: the infectious class is decomposed into several sub-classes, with an identical rate of transition between these classes chosen to retain the same expected infectious period. Such an approach maintains the Markov property of the underlying model and thus the applicability of the methodology outlined above (see for example [39] and [40]).

When comparing results of gamma-distributed to the traditional exponentially-distributed periods, we consider two cases: i) in the first we hold the transmission rate parameter 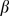 constant; ii) whilst in the second we hold the probability of (initial) transmission, 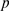, constant. The latter is evaluated as follows: Consider a household with one individual initially infected and all other members susceptible to infection, with transmission rate 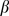, mean infectious period 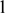, and gamma-distributed recovery time of order 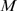 (referring to dividing the infectious period into 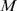 sub-classes, 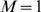 is exponentially distributed and the limit 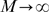 gives a constant time to recovery). The probability that the initial infective infects at least one other individual before recovering is
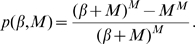
Note, 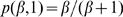 and 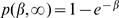. We consider holding 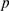 constant (via changing 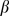, to 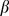) as we change 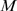 because this is similar to holding constant the *secondary attack rate* (we discuss this further in the Supporting Information File S1), a quantity that is often measured during statistical analysis of household data [49].

## Results

**Figure 1:**
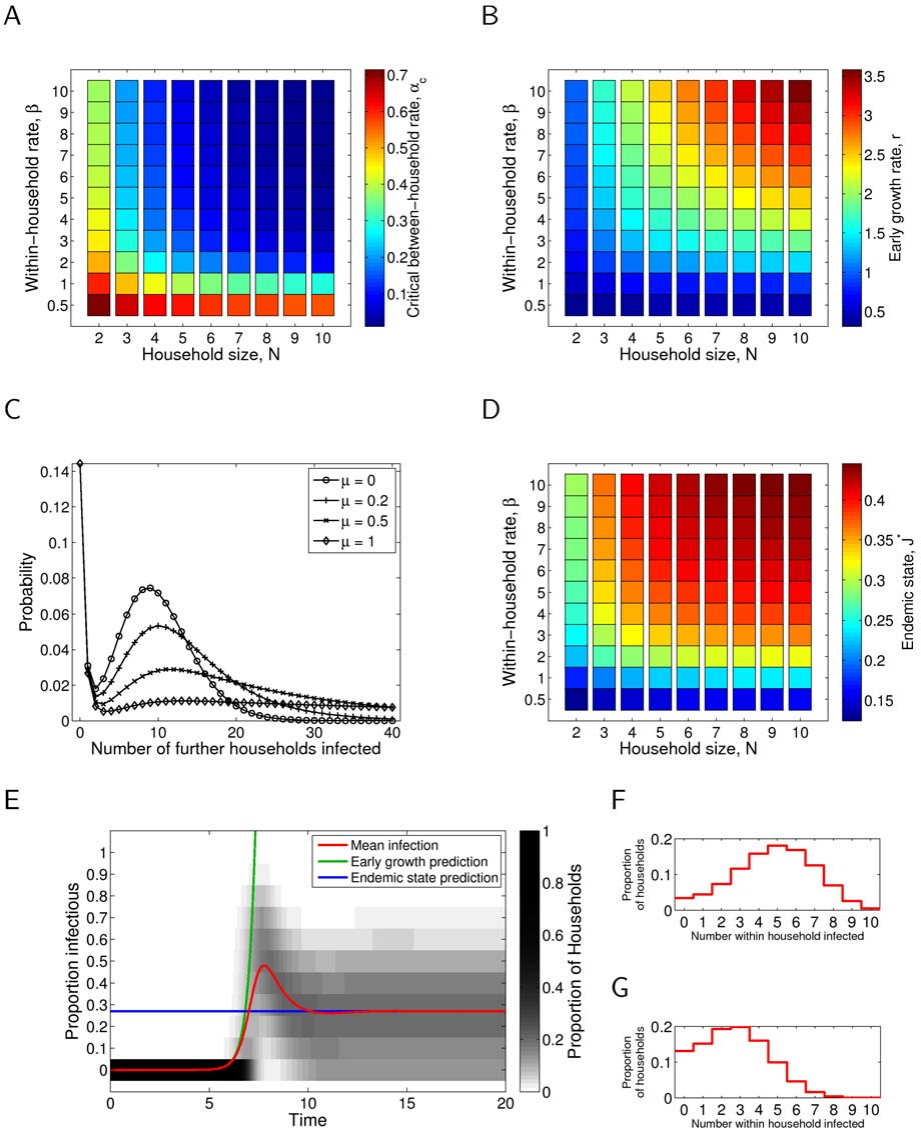
Epidemiological quantities as defined in the main text for the exponential (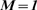) infectious period distribution. (A) Critical transmission 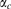, with rate of waning immunity 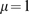. (B) Early growth rate 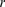, with rate of waning immunity 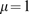 and between-household transmission parameter 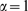. (C) Offspring distribution, with within-household transmission rate parameter 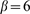, house size 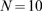 and between-household transmission parameter 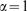. (D) Endemic infection 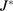, with rate of waning immunity 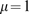 and between-household transmission parameter 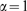. (E) Transient dynamics, with within-household transmission rate parameter 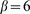, house size 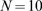, rate of waning immunity 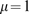 and between-household transmission parameter 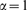. (F) As in E at peak prevalence. (G) As in E at endemic prevalence.

Figure 1 shows the critical level of between-household transmission, 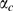, required to sustain an epidemic (Plot 1A); the early growth rate 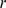 (Plot 1B); the offspring distribution (Plot 1C); the endemic prevalence proportion 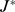 (Plot 1D); and the transient dynamics of infection (Plot 1E; see 1F and 1G also), all for the case of exponentially distributed infectious period (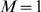). The precise parameter values are provided in the Figure caption; other rate parameter values are presented in the Supporting Information File S1.

Examining the critical level of between-household transmission (Plot 1A) indicates that two factors contribute to the success of an infection. For large household sizes, the main determinant of epidemic success is whether within-household transmission (governed by 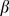) can produce an epidemic within the household – if it can, then the final size within households is relatively large and so relatively small levels of between-household transmission, 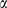, can sustain an epidemic. For smaller households of size 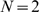 and 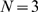, this is not seen, and appreciable between- and within-household transmission is always necessary to sustain an epidemic.

The endemic prevalence of infection (when between-household transmission rate 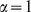; Plot 1D) is again predominately determined by within-household transmission, 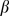, for larger households, with household size, 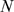, only having a major impact when it is below size 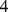. However, unlike the critical level of transmission (Plot 1A) and early growth rate (Plot 1C), varying the rate of waning immunity, 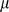, has a significant effect in terms of absolute prevalence 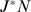, which reduces as the rate of loss of immunity 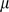 is reduced, and vanishes in the absence of waning immunity (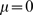); see Supporting Information File S1.

The early growth rate 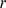 (with between-household transmission 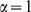; Plot 1B) varies with both within-household transmission, 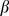, and household size, 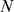, and is only weakly affected by waning immunity, 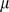 (see Supporting Information File S1). We observe that the critical between-household transmission rate (Plot 1A) and the endemic prevalence of infection (Plot 1D) show far greater saturation with both household size, 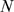, and within household transmission rate, 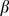, compared to the early growth rate (Plot 1B). This is because the critical transmission rate, 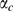, and the endemic prevalence of infection, 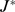, depend on the number of cases produced over one generation (which rapidly saturates as susceptibles within the household get infected), whereas the early growth rate, 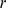, depends on the instantaneous transmission rate from infected individuals (which is less influenced by households reaching saturation).

The offspring distribution (Plot 1C) shows a significant probability that a newly infected household will fail to infect any further households; often because the infection fails to spread within the household. This failure probability is relatively unaffected by the waning immunity rate, 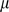; whereas increasing 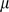 leads to an increased probability of generating very large numbers of secondary cases. The bimodality of these distributions is a qualitative difference from distributions considered in detail at the individual level [50], [51] and therefore can be expected to lead to very different stochastic invasion and persistence properties.

The complete model dynamics at a given parameter set (Plot 1E) demonstrates that our methods for calculating early growth (green line) and the endemic state (blue line) are sound, and also that the proportion of households with a given prevalence assumes a unimodal distribution around the mean, with significant variance.

**Figure 2:**
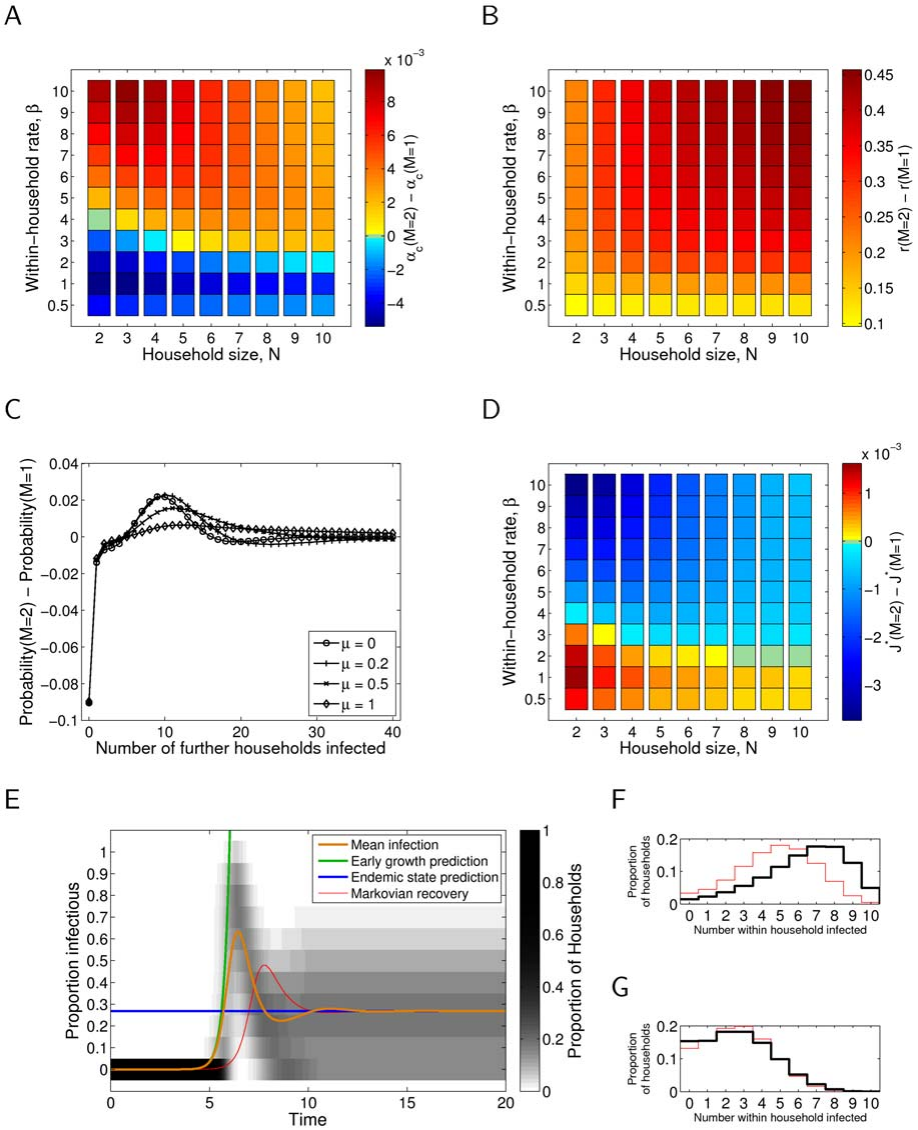
Epidemiological quantities as defined in the main text for the gamma (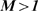) infectious period distribution, compared to the exponential (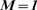) results, where within household transmission rate parameter, 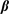, is held constant. (A) Critical transmission difference 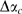, with rate of waning immunity 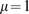; 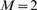. (B) Early growth rate difference 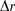, with rate of waning immunity 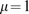 and between-household transmission parameter 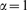; 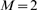. (C) Offspring distribution difference, with within-household transmission rate parameter 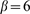, house size 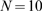 and between-household transmission parameter 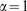; 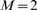. (D) Endemic infection difference 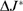, with rate of waning immunity 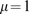 and between-household transmission parameter 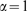; 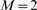. (E) Transient dynamics, with within-household transmission rate parameter 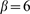, house size 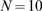, rate of waning immunity 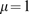 and between-household transmission parameter 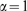; 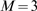; exponential (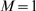) results are shown as a thin red line for comparison. (F) As in E at peak prevalence. (G) As in E at endemic prevalence.

**Figure 3:**
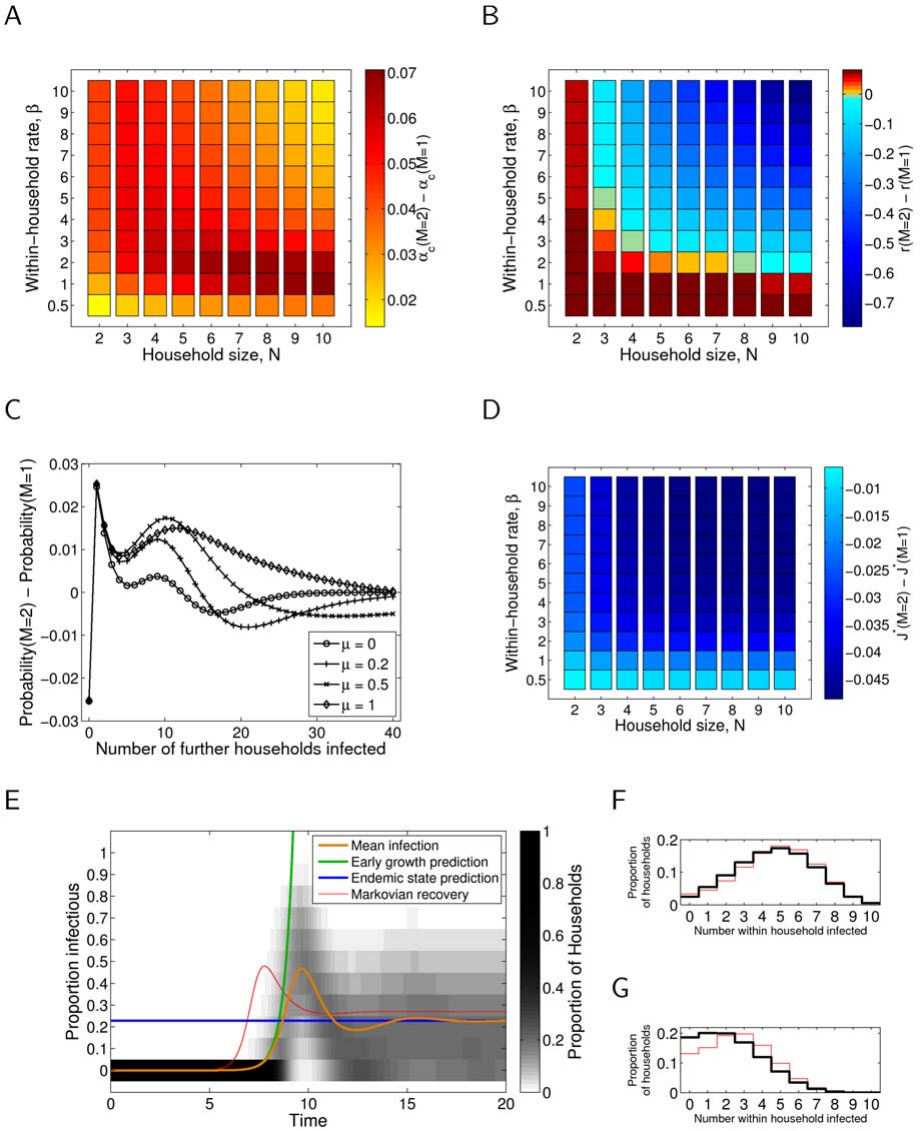
Epidemiological quantities as defined in the main text for the gamma (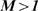) infectious period distribution, compared to the exponential (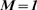) results, where probability of transmission, 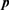, is held constant. (A) Critical transmission difference 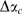, with rate of waning immunity 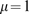; 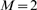. (B) Early growth rate difference 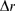, with rate of waning immunity 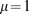 and between-household transmission parameter 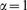; 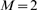. (C) Offspring distribution difference, with within-household transmission rate parameter 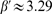 (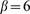), house size 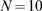 and between-household transmission parameter 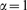; 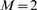. (D) Endemic infection difference 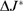, with rate of waning immunity 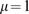 and between-household transmission parameter 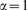; 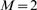. (E) Transient dynamics, with within-household transmission rate parameter 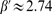 (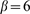), house size 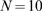, rate of waning immunity 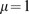 and between-household transmission parameter 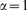; 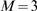; exponential (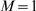) results are shown as a thin red line for comparison. (F) As in E at peak prevalence. (G) As in E at endemic prevalence.

Figures 2 and 3 essentially repeat the evaluation of each of the epidemiologically relevant quantities for the case of gamma distributed infectious period of order 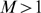, with 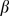 and 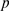 held constant, respectively. In panels (A), (B), (C) and (D) the change in the quantity (respectively, 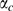, 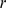, probability of offspring number, and 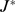) with respect to the assumption of an exponential infectious period has been presented; likewise, in (E), the mean prevalence curve and, in (F) and (G), the full distribution of prevalence, under the assumption of an exponential infectious period have been superimposed for reference.

With respect to the critical transmission rate, 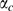, the early growth rate, 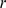, and the endemic prevalence of infection, 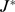, it can be seen that both the magnitude and direction of change can differ depending upon whether the transmission parameter 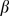 or the probability of transmission, 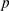, is held constant. With respect to these quantities, holding 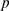 constant generally results in more significant changes. Furthermore, in general, a higher level of between-household transmission, 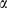, is required to sustain an epidemic, and thus the endemic prevalence of infection, 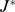, with 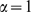 is generally reduced. The early growth rate increases if 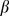 is held constant (Plot 2B) and generally decreases if 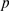 is held constant (Plot 3B).

With respect to the offspring distributions (C), once again the incorporation of gamma-distributed infectious period, and choice of what is held constant between epidemics, has a significant impact. In both cases the probability of no secondary households infected decreases, and by a substantial margin in the case of 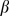 held constant. In the 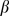 constant case the probability of a small number of secondary infections also decreases, whilst in the 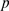 constant case this is not seen. In both cases there is a decrease in the tail of the distribution, corresponding to reduced probability of a large number of secondary infections, and the major increase in probability mass occurs in the vicinity of the second peak of the exponential offspring distribution case. Despite these changes, for this parameter set, the bimodal feature of these distributions remains.

Finally, we consider the influence of gamma-distributed infectious period, and what is held constant, by studying the full dynamics of infection. Holding 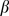 constant (Plot 2E) results in an earlier epidemic with a larger peak infection when compared to the exponential case, whilst holding 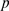 constant (Plot 3E) results in an epidemic with similar, but slightly reduced, peak incidence but slower take-off and hence delayed peak. Also interestingly, the incorporation of gamma-distributed infectious period results in a slight oscillatory approach to endemicity, with mean infection dropping below the endemic prevalence following the peak, and slightly overshooting the endemic level again before converging to equilibrium.

## Discussion

In this paper, we have focused on appropriate numerical methods for efficient calculation of epidemiologically relevant quantities in households models obeying an *SIRS* disease paradigm. Solutions of the kind we have found provide a useful bridge between formal work [36] and individual-based simulation [24], allowing us to study the effects of finite size, stochasticity, and infectious and recovered period distributions at the household level while still in the infinite-size limit at the population level. This has allowed us to quantify some basic behaviours of household structured dynamics; for example the impacts of varying within-household and between-household transmission rates and the role of waning immunity.

In essence the behaviour of the households model can be explained by two different processes. Firstly, compared to purely individual-based transmission (at rate 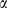) the action of within-household transmission (at rate 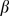) is to amplify the infection; so households act as amplifiers for the general transmission process. Secondly, the clustered network structure within a household leads to rapid depletion of the locally susceptible population and hence saturation of amplification effect (and other household properties) as the within-household transmission rate increases. Using our methods, a full sweep of parameter space (as provided in Supporting Information File S1) requires only a few seconds of desktop processor time and so comprehensive sensitivity analysis around a region of parameter space relevant to a given applied problem is also readily obtained. In addition, such methods are able to rapidly calculate likelihood values for any given observations, leading to efficient methods of parameter estimation from household-structured data.

Our results additionally provide one of the first explicit studies of the impact of gamma-distributed infectious periods on dynamics at the household level. Biologically significant effects arise from this change in infectious period distribution, with both the magnitude and direction of these effects varying depending on which parameters are held constant when comparing models. This link between which quantities are observed and therefore which model parameters are held constant, is likely to have significant impact during the early stages of a disease outbreak in determining an appropriate response.

The methodology used to consider gamma-distributed infectious periods can be straightforwardly extended to include latent and prodromal stages of infection. For homogeneously mixed models, the most common such addition is an ‘exposed’ class through which infected individuals pass before becoming fully infectious, leading to the standard *SEIR* model. This does not alter threshold and final size behaviour compared to the *SIR* model, but can cause a highly significant reduction of the early growth rate and modification of other features of the transient dynamics, which we would also expect to see in household models.

We also believe that the epidemiological consequences of the shape of the household offspring distribution warrant further consideration, as has been done for individual-level offspring distributions [50], [51]. The ability to construct this distribution through the solution of sets of linear equations offers the possibility of deriving such a distribution, and therefore a greater understanding of stochastic invasion and persistence, for a range of more complex disease natural histories.

Methodologically, we hope that the reduction of many problems in household epidemic theory to solving a set of linear equations through the theory of path integrals for Markov chains will be of significant use, and have made MATLAB code available to encourage this in the Supporting Information File S2. It is important to note that the consideration of distributions of household sizes is simply done within our framework, but is not included in this work since its main impact is known, from theoretical work, to be on control strategy rather than dynamics. While it could be argued that the methodologies presented here simply allow us to produce the same results more quickly than by using direct integration, numerically efficient algorithms can open up problems to analysis that are currently unsolvable. One example of this would be to consider epidemiological dynamics of sub-populations much larger than households, either ecologically-motivated or as a simplification of large-scale structured models of human and animal disease.

## Supporting Information

File S1 Calculation of disease dynamics in a population of households. In this Supporting Information File S1, we include additional technical discussion, and supplementary figures.
(0.08 MB PDF)

File S2 Calculation of disease dynamics in a population of households. In this Supporting Information File S2 we provide MATLAB code.
(0.01 MB ZIP)
