# Supplementary material for: Calculation of Disease Dynamics in a Population of Households
Source: PLoS One. 2010 Mar 18;5(3):e9666. doi: 10.1371/journal.pone.0009666 (PMC2841206; doi:10.1371/journal.pone.0009666)
Supplement: File S1 — Calculation of disease dynamics in a population of households. In this Supporting Information File S1, we include additional technical discussion, and supplementary figures. (0.08 MB PDF) [file pone.0009666.s001.pdf]

Calculation of disease dynamics in a population of households: Supporting Information File S1

J.V. Ross, T. House and M.J. Keeling

In this supporting information, we include additional technical discussion and supplementary figures.

SAR versus p

In the main text, we held constant the probability that the initial infective infects at least one other individual before recovering, which for exponentially distributed recovery at unit rate is

$$p = \frac{\beta}{\beta + 1}.$$

This is not quite the same as the (expected) secondary attack rate (SAR), which is the (expected) proportion of secondary cases actually caused by the primary case—although different definitions do exist in the literature.

As an example of this difference, we consider a three-person household with exponentially distributed *SIR* dynamics and one individual initially infected. The possible states of this system are shown in Figure S1, with numbers corresponding to the number of secondary infections due to the primary case. The essential point is that once a second household member has become infected, he/she and the initially infected individual are both 'competing' to infect the third member, meaning that the SAR is

$$\mathrm{SAR} = p\left(1 - \frac{\beta^2}{2(\beta + 1)(2\beta + 1)}\right) \times 100\%.$$

Essentially, each possible proportion of secondary infections due to the primary case must be weighted by the probability of the occurrence of that event and summed. The analytic calculation of this quantity, while possible for the three-person household by explicit construction of a Markov chain, becomes very time-consuming for larger households and gamma-distributed recovery times. However, the SAR and p are conceptually similar, and holding p constant should have similar consequences to holding the SAR constant.

Varying waning immunity

The Figures S2–S4 are all for the case of exponentially distributed infectious period ($M = 1$), and provide the critical level of within-household tranmission, α_c, the early growth rate, r, and the endemic prevalence of infection, $J^* = I^*/N$, as presented in the main text for a wider range of rates of waning immunity, μ: from $\mu = 0$ corresponding to *SIR* dynamics, to $\mu \to \infty$ corresponding to *SIS* dynamics.

Increasing μ results in a decrease in α_c, as waning immunity increasingly replenishes the pool of susceptibles. The early growth rate, r, increases, slightly, with increasing μ, as individuals spend less time recovered; however, being the *early* growth, the impact is not significant unless μ is very large.

Finally, and for the same reason, the endemic prevalence of infection increases with increasing μ; obviously, in the case $\mu = 0$ (*SIR*) no infection is present in the long-term.

Supplementary Figures

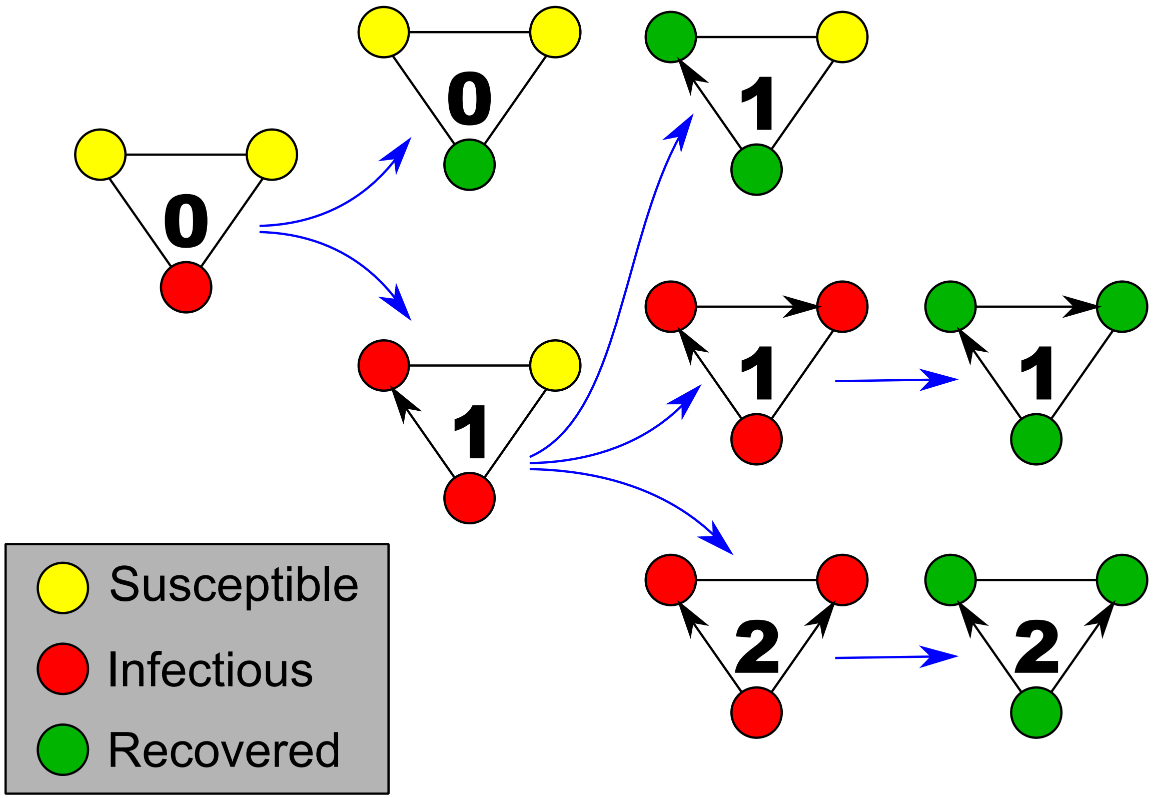

Figure S1: Possible routes of infection through a three-person household starting with one initial infected individual. Blue arrows show transitions between household states, with black arrows showing the transmission of infection within the household. Black figures represent the number of infections due to the original infected. Some intermediate recovered states are omitted for clarity.

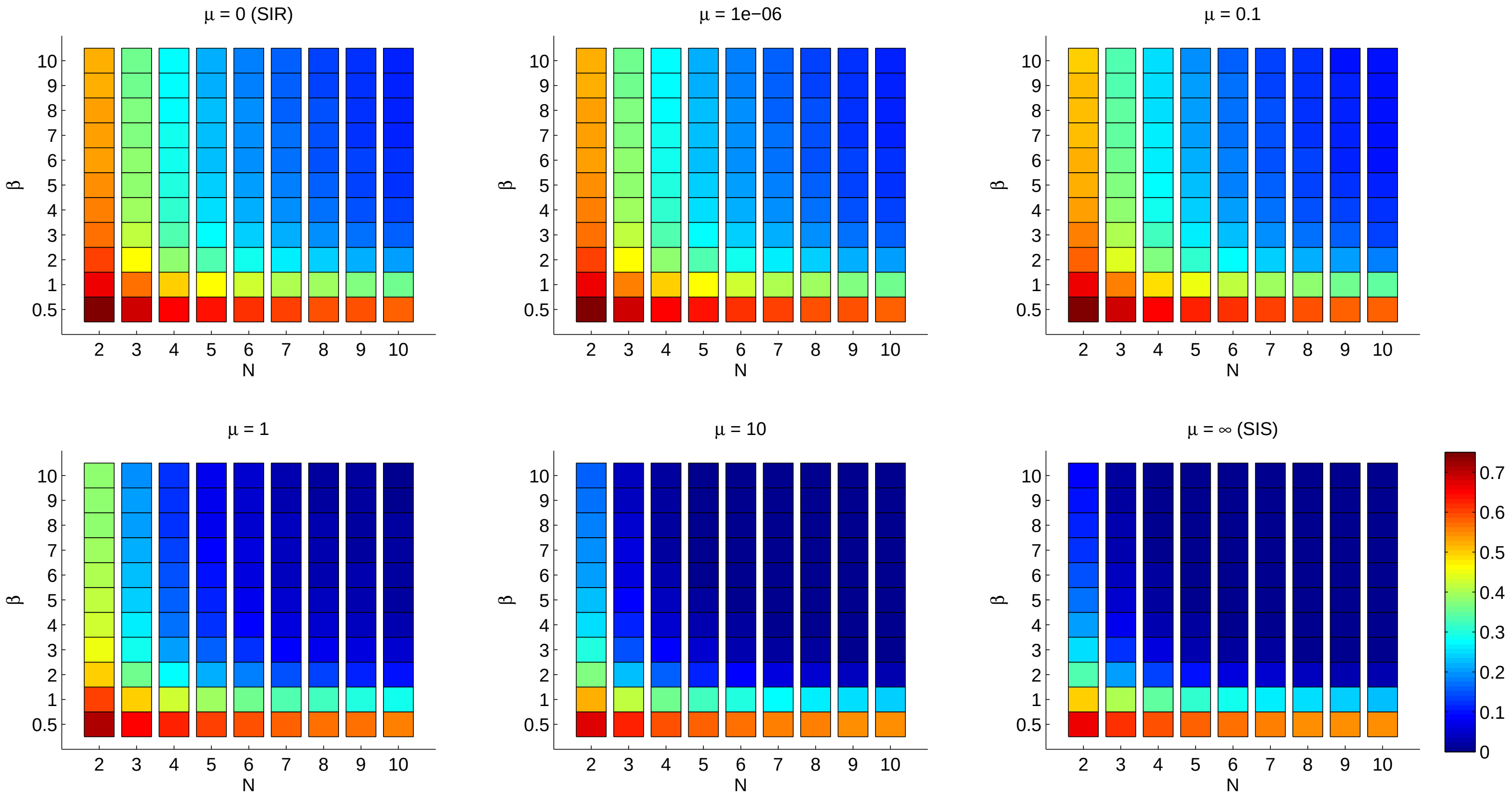

Figure S2: Full sweep of parameters for critical α_c values.

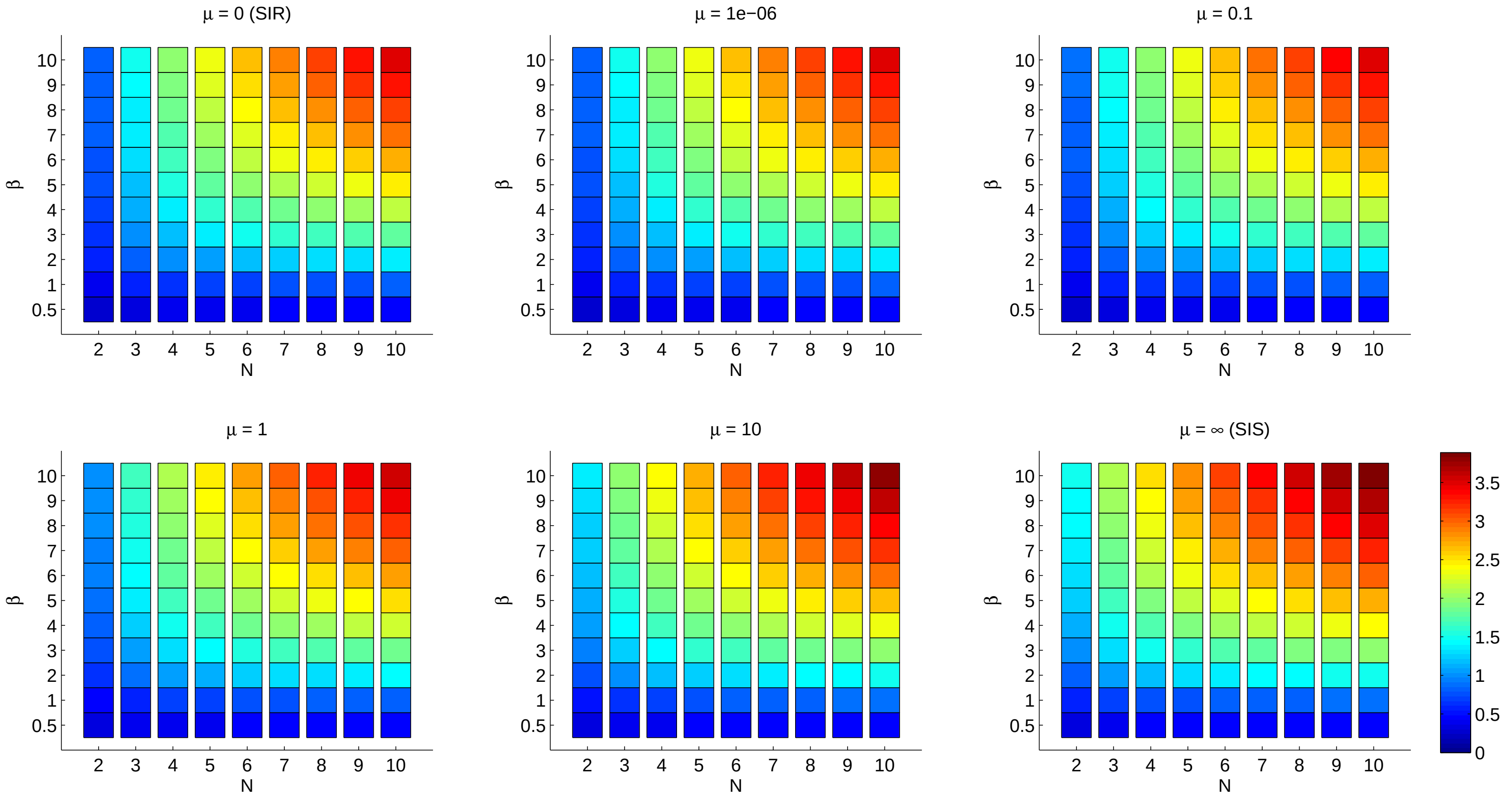

Figure S3: Full sweep of parameters for early growth rates, r, at $\alpha = 1$.

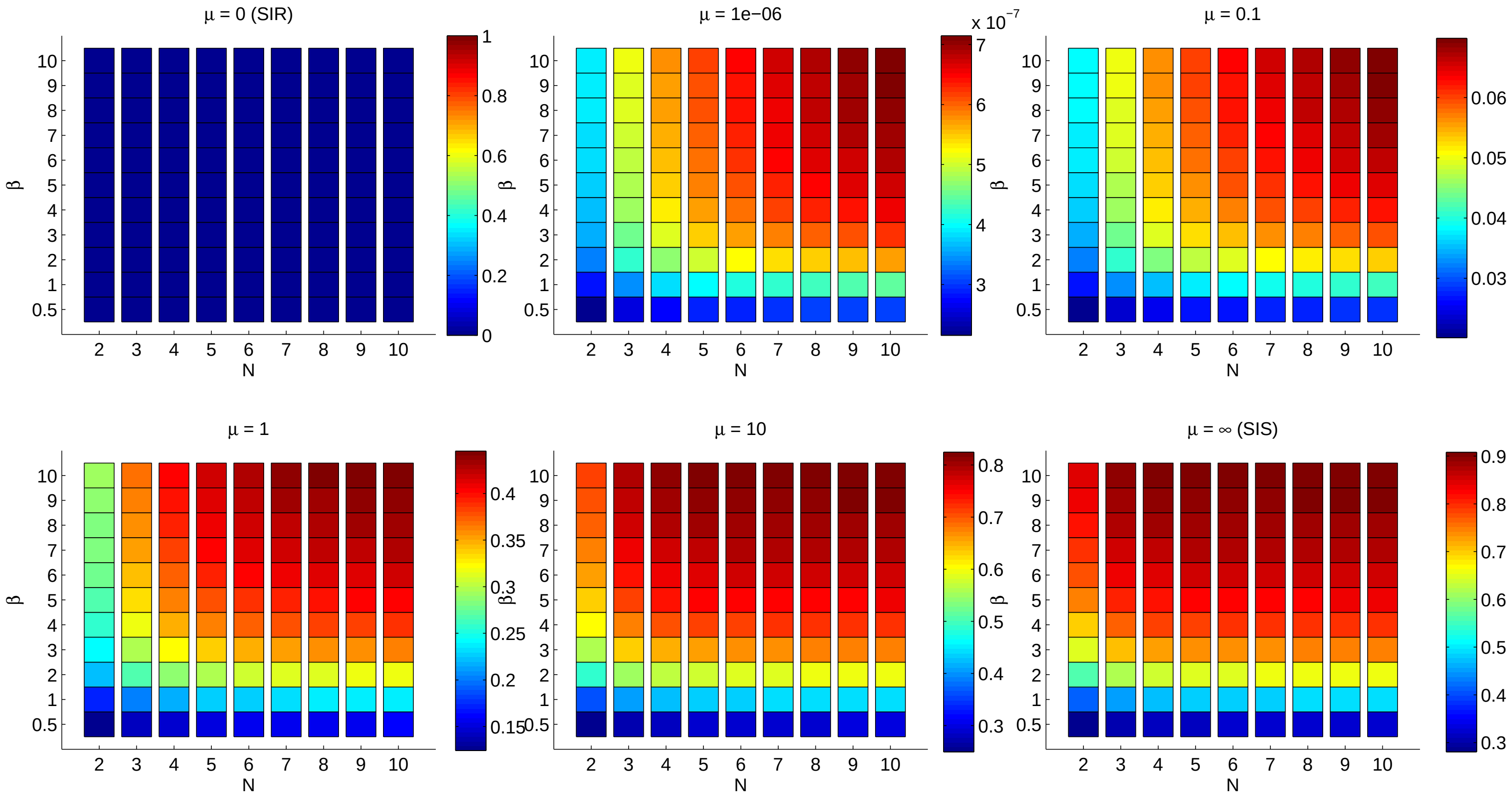

Figure S4: Full sweep of parameters for endemic states, J^*, at $\alpha = 1$.
